# Effects of the Chinese Herbal Formulation (*Liu Wei Di Huang Wan*) on the Pharmacokinetics of Isoflavones in Postmenopausal Women

**DOI:** 10.1155/2015/902702

**Published:** 2015-06-04

**Authors:** Wirin Limopasmanee, Sunee Chansakaow, Noppamas Rojanasthien, Maleeya Manorot, Chaichan Sangdee, Supanimit Teekachunhatean

**Affiliations:** ^1^Department of Pharmacology, Faculty of Medicine, Chiang Mai University, Chiang Mai 50200, Thailand; ^2^Department of Pharmaceutical Sciences, Faculty of Pharmacy, Chiang Mai University, Chiang Mai 50200, Thailand; ^3^Center of Thai Traditional and Complementary Medicine, Faculty of Medicine, Chiang Mai University, Chiang Mai 50200, Thailand

## Abstract

A combination of soy isoflavones and *Liu Wei Di Huang Wan* (LWDHW) is potentially effective for postmenopausal women with intolerable vasomotor episodes who are not suitable candidates for hormonal therapy. The objective of this open-label, three-phase, crossover study was to determine the influence of both single and multiple oral doses of LWDHW on isoflavone pharmacokinetics in healthy postmenopausal women. Eleven subjects were assigned to receive the following regimens in a fixed sequence with washout periods of at least one week: Phase A, a single oral dose of soy milk; Phase B, a single oral dose of soy milk coadministered with LWDHW; and Phase C, multiple oral doses of LWDHW for 14 days followed by a single oral dose of soy milk. Blood samples were collected and mixed with *β*-glucuronidase/sulfatase to hydrolyze isoflavone conjugates to their respective aglycones (i.e., daidzein and genistein) and were determined using high performance liquid chromatography. The pharmacokinetic parameters analyzed were maximal plasma concentration (*C*_max_), time to reach peak concentration (*T*_max_), area under the plasma concentration-time curve (*AUC*), and half-life (*t*_1/2_). The results found no statistically significant differences in pharmacokinetic parameters of daidzein and genistein among the three regimens.

## 1. Introduction

Menopause is a period of time in a woman's life defined as the cessation of menstruation for more than 12 months and involves permanent failure of ovarian function secondary to depletion of the follicular pool. This commonly occurs in Asian women who are in their mid-to-late 40s [1, 2]. Vasomotor episodes are the most characteristic manifestation of the menopause. These episodes are described as hot flushes (the sensations of warmth, usually felt on the chest, neck, and face), hot flashes (episodes with sweating, sometimes followed by a chill), and night sweats. However, the terms “hot flushes” and “hot flashes” are often used interchangeably [1]. The proportion of women who report hot flashes may be as great as 80% in western countries but as low as 10% in some East Asian countries. Hormone therapy (HT) diminishes vasomotor symptoms in a dose-dependent fashion. However, the use of estrogen alone in HT increases the risk of endometrial hyperplasia and cancer, while continuous use of combined HT (estrogen plus progestin) may increase the risk of coronary heart disease, breast cancer, stroke, and venous thrombosis [3–5]. Concern over the potential adverse effects of HT leads many women to look for natural alternatives that would provide benefits comparable to those of estrogen but without serious complications.

Soy isoflavones, known as phytoestrogens, have been shown to reduce vasomotor symptoms significantly without an adverse effect on the endometrium or vagina [6, 7]. In addition, they are considered beneficial for reducing the incidence of cardiovascular disease, hormone-dependent breast cancer, colon cancer, and osteoporosis [8–11]. Likewise, Traditional Chinese Medicine (TCM) is one attractive option for alleviating menopausal symptoms. According to TCM, vasomotor symptoms (especially hot flushes and night sweats) are caused by Kidney Yin Deficiency. The Chinese herbal formulation of choice for nourishing Kidney Yin Deficiency is “*Liu Wei Di Huang Wan*” (LWDHW) or “Six-Ingredient Pill with Rehmannia.” This formulation consists of* Shu Di Huang* (Radix Rehmanniae Preparata),* Shan Zhu Yu* (Fructus Corni),* Shan Yao* (Rhizoma Dioscoreae),* Ze Xie* (Rhizoma Alismatis),* Mu Dan Pi* (Cortex Moutan), and* Fu Ling* (Poria). A modified formulation of LWDHW has been shown to be significantly effective in reducing the number of hot flushes without serious adverse reactions [12, 13]. LWDHW, in addition to being a popular Chinese herbal formula used in the treatment of vasomotor symptoms, is also used in the treatment of other medical conditions such as diabetes [14], hypertension [15], and disorders of the immune system [16].

As both soy isoflavones and LWDHW are potentially effective for management of vasomotor symptoms, the combination of both modalities to maximize their efficacy is considered a plausible attractive option, especially for some perimenopausal or postmenopausal women who experience intolerable vasomotor episodes but are not suitable candidates for HT; however, no study of the pharmacokinetic interaction between the two interventions has been done. The main objective of this study was to determine the influence of single and multiple oral doses of LWDHW on isoflavone pharmacokinetics in healthy postmenopausal women.

## 2. Materials and Methods

### 2.1. Study Design

This study was an open-label, fixed-sequence, three-phase, crossover study, with washout periods of at least 1 week. The study was carried out at the Clinical Pharmacology Unit, Faculty of Medicine, Chiang Mai University. The study protocol was approved by the Human Research Ethics Committee of the Faculty of Medicine, Chiang Mai University, and it complied with the Helsinki Declaration. The details of the study were clearly described to all participants and a signed informed consent was voluntarily obtained from them prior to their participation in the study.

### 2.2. Participants

The sample size calculation was based on the assumptions that the content of absorbed isoflavones (equivalent to the area under the plasma concentration-time curve or* AUC*) would be the main efficacy criterion, and the mean difference in* AUC* following concurrent administration of LWDHW and soy milk (*μ*_2_) in comparison to* AUC* following consumption of soy milk alone (*μ*_1_) was assumed to be 0 (i.e., *μ*_2_ − *μ*_1_ = 0). The noninferiority margin (*δ*) was estimated to be 20%, and the standard deviation (*σ*) was expected to be 18.5. By using the following formula for noninferiority trials [17], the required sample size to achieve an 80% power (*β* = 0.2) at *α* = 0.05 for detecting such difference was 11 participants. Consider

(1) $$\begin{array}{c} n_{1}=n_{2}=\frac{2{\left(z_{\alpha }+z_{\beta }\right)}^{2}\sigma^{2}}{{\left(\mu_{2}-\mu_{1}-\delta \right)}^{2}}. \end{array}$$

Eleven postmenopausal Thai women, aged at least 45 years with cessation of menstruation for at least 12 months and serum follicle-stimulating hormone concentration >30 IU/L, were enrolled in this study. Their body mass index (BMI) was between 18–25 kg/m^2^. Each participant underwent a physical and blood examination including complete blood count (CBC), blood urea nitrogen (BUN), creatinine (Cr), and liver function test (LFT) to confirm their health status. Postmenopausal women with a prior history of malignancy or renal, liver, pulmonary, cardiovascular, or gastrointestinal diseases were excluded from the study as were women with a known history of cigarette smoking, alcoholic consumption, substance abuse, or addiction. Other exclusion criteria were the use of antibiotics, laxatives, prebiotics, probiotics, synbiotics, other medications, and food supplements within the preceding four weeks. All participants were requested to abstain from soy-food products, other medications, herbal or food supplements, caffeine-containing beverages, prebiotics, probiotics, and synbiotics during all study periods.

### 2.3. Dosage and Drug Administration

#### 2.3.1. Soy Preparation

The soy preparation used in this study was UHT soy milk (V-Soy, expiration date 5 September 2014, manufactured by Green Spot Co., Ltd, Bangkok, Thailand). The mean isoflavone content of daidzin, genistin, daidzein, and genistein was 37.74 ± 1.56, 36.95 ± 1.50, 11.21 ± 0.40, and 6.82 ± 0.24 mg/375 mL, respectively.

#### 2.3.2. LWDHW

The LWDHW used in this study was manufactured by the Faculty of Pharmacy, Chiang Mai University, Thailand. It consisted of 24% Radix Rehmannia Preparata, 12% each of Fructus Corni and Rhizoma Dioscoreae, 9% each of Rhizoma Alismatis, Cortex Moutan, and Poria as well as 25% honey. All six crude drugs were purchased from Vejpong Pharmacy Co., Ltd (Bangkok, Thailand). Raw materials used in this formula were evaluated following* Chinese Pharmacopoeia 2005* [18]. Chemical control of LWDHW was analyzed by high performance liquid chromatography coupled with photodiode array (HPLC-PDA) using loganin as the active marker. The standardized crude drugs of LWHDW were ground into a fine powder and mixed with heated honey to make boluses approximately 4 mm in diameter. Quality control of the honey pill (i.e., assessment of weight variation, disintegration, moisture content, and microbial contamination) was conducted following WHO guidelines on good manufacturing practices (GMP) for herbal medicines [19].

#### 2.3.3. Drug Administration

**Figure 1:**
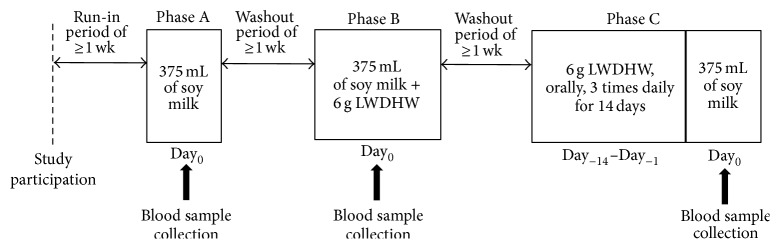
Administration of soy milk and/or LWDHW in Phases A, B, and C (treatment in each phase was given in a fixed-sequence approach). Phase A, a single oral dose of 375 mL of soy milk; Phase B, a single oral dose of 375 mL of soy milk coadministered with a single oral dose of 6 g LWDHW; and Phase C, an oral administration of 6 g LWDHW three times daily for 14 days followed by a single oral dose of 375 mL of soy milk on the next day. Blood sample collection: before administration of soy milk and at 0.5, 1, 2, 4, 6, 8, 10, 12, 24, and 32 hours after administration of soy milk.

This study consisted of three phases in a fixed-sequence approach (Phases A, B, and C) as shown in Figure 1. After a run-in period of at least one week, participants were assigned to receive a single oral dose of 375 mL of soy milk (Day_0_ of Phase A). After a washout period of at least one week, participants underwent Phase B during which a single oral dose of 6 g LWDHW was coadministered with a single dose of 375 mL of soy milk (Day_0_ of Phase B). Subsequently, participants underwent a washout period of at least one week and continued to receive treatment in Phase C during which 6 g LWDHW was administered orally three times daily (approximately one hour before meals) for 14 days, followed by a single oral dose of 375 mL of soy milk on the next day (Day_0_ of Phase C). In each phase of this study, soy milk was orally administered in the morning after an overnight fast of at least eight hours. Participants were fasted for two hours and were requested to remain in an upright position for four hours following soy milk consumption. Water, lunch, and dinner were served at 2, 4, and 10 hours, respectively, following administration of the soy milk. Identical isoflavone-free diets and beverages were served to all participants during the three phases of this study. All participants were requested to refrain from isoflavone-containing products as well as some specific foods and beverages (e.g., soy milk, tofu, prebiotics, probiotics, synbiotics, alcohol, caffeine beverages, etc.) throughout the study period. During each phase, blood samples were collected at different time points (see below).

### 2.4. Collection of Blood Samples

On Day_0_ of each phase, venous blood samples (6 mL) for quantification of isoflavone levels were collected from the forearm by venipuncture through an indwelling intravenous catheter prior to an administration of soymilk and then at 0.5, 1, 2, 4, 6, 8, 10, 12, 24 and 32 hours after the dose. Blood samples were collected in heparinized vacutainer tubes and were centrifuged at 2500 rpm for 20 minutes. Thereafter, plasma was separated and immediately maintained at −70°C in a freezer until analyzed.

### 2.5. Determination of Plasma Isoflavones by High Performance Liquid Chromatography with UV-Diode-Array Detection (HPLC-UV)

#### 2.5.1. Sample Preparation

Plasma sample preparation was performed as described by Timan et al., 2014 [20]. Specifically, 250 *μ*L of plasma was mixed with 150 *μ*L of an enzyme mixture of *β*-glucuronidase/sulfatase in order to hydrolyze glucuronide and sulfate metabolites to daidzein and genistein. The enzyme mixture consisted of 0.1 g ascorbic acid in 10 mL of 0.1 M sodium acetate buffer containing 0.01 g ethylenediaminetetraacetic acid (EDTA) and 250 *μ*L of *β*-glucuronidase/sulfatase obtained from* Helix pomatia* (SIGMA G-0876). To complete the hydrolysis of isoflavone conjugates, plasma samples with the enzyme mixture were heated in a water bath at a temperature of 37°C for 15–18 hours and then allowed to cool to room temperature. Thereafter, 10 *μ*L of 50,000 ng/mL fluorescein in 80% methanol was used as the internal standard (IS) and added to the plasma sample, followed by 1,000 *μ*L of 1% acetic acid in acetonitrile to deproteinize the samples which were then vortex mixed for 30 seconds. The samples were centrifuged at 14,000 rpm for 10 minutes to separate the supernatant and were then evaporated to dryness by a vacuum concentrator at 60°C for three hours. Subsequently, 50 *μ*L of mobile phase B (described below) was added to reconstitute the residue, and 5 *μ*L of the reconstituted sample was injected into the HPLC system.

#### 2.5.2. HPLC-UV Conditions

The determination of isoflavone levels in plasma was performed by HPLC-UV as described by Timan et al., 2014 [20]. The HPLC assay was conducted using a UV detection wavelength of 259 nm. The chromatography system consisted of a 5-*μ*m C-18 column equipped with a guard column (Inersil ODS-3, GL Sciences, Japan). The mobile phases used in this HPLC condition were mobile phase A and B consisting of 60 mM ammonium acetate in deionized water/methanol/acetonitrile in the proportions of 250/45/45 (v/v/v) and 250/220/255 (v/v/v), respectively. Both mobile phases contained 250 *μ*L of 1.44 mM sodium dodecyl sulfate and 30 *μ*L of perchloric acid. A chromatographic separation was scheduled using a gradient elution of 100% A at 0–3 min, 70 : 30 with A : B at 3.1–9 min, 20 : 80 at 9.1–18 min, and 85 : 15 at 18.1–25 min, respectively. The system was operated with flow rate of 1 mL/min at 25°C. The chromatogram revealed that retention times of IS, daidzein, and genistein were 11.234, 14.357, and 16.389 minutes, respectively.

#### 2.5.3. Assay Validations

Calibration curve plots demonstrated that the linear regressions of daidzein and genistein concentrations ranged from 37.5, 75, 150, 300, 600, 1200, and 2400 ng/mL versus peak height ratios of isoflavones and IS gave coefficients of determination (*r* ^2^) greater than 0.9997.

For intraday precision, five replicated samples from each of three quality control (QC) samples (112.5, 1125, and 2250 ng/mL) were quantified with a single calibration curve, while determinations of five replicated samples of the respective QC samples using five concurrent standard calibration curves on five dependent days were conducted to evaluate interday precision.

Precision was shown as the percentage coefficient of variation (% CV) calculated as

(2) %  CV=Standard  deviationMean  value  of  isoflavone  concentrations  in  plasma×100.

The inaccuracy was presented as the percentage of deviation (% deviation) calculated as

(3) %  Deviation=Measured  concentration−Spiked  concentrationSpiked  concentration×100.

The % CV of intraday precision for the three respective QC samples (112.5, 1125, and 2250 ng/mL) were 3.41, 1.04, and 1.82% for daidzein and 2.71, 0.78, and 1.15% for genistein. For interday precision, % CV for the three QC samples were 2.46, 3.53, and 4.08% for daidzein and 5.93, 3.25, and 3.24% for genistein.

The percentage deviations of intraday assay for the three QC samples of daidzein were −3.58, −1.10, and −4.34%, and those of genistein were 9.13, −4.61, and −4.95%, while the percentage deviations of interday assay of daidzein were −6.73, 1.46, and −1.95%, and those of genistein were 2.16, −4.62, and −5.02%. The mean recovery values (± standard deviation) of daidzein, genistein, and IS were 93.45 ± 3.55%, 90.57 ± 5.94%, and 97.69 ± 2.38%, respectively.

### 2.6. Pharmacokinetics Parameters and Statistical Analysis

#### 2.6.1. Pharmacokinetics Parameters

The pharmacokinetic parameters analyzed in this study were maximal plasma concentration (*C*_max_), time to reach the peak concentration (*T*_max_), the area under the plasma concentration-time curve from time 0–32 hours and 0–*∞* hours (*AUC*_0–32_ and *AUC*_0–*∞*_), and the terminal half-life (*t*_1/2_). These parameters were determined by noncompartmental analysis. The *C*_max_ and *T*_max_ were acquired by direct visual inspection of each subject's plasma concentration-time curve profile. The *t*_1/2_ was calculated as the ratio of 0.693 to the terminal rate constant (*k*_*e*_), while *k*_*e*_ was obtained from the terminal slope on the semilog concentration versus time plot. The trapezoidal rule was used for the *AUC*_0–32_ calculation. The *AUC*_32–*∞*_ was extrapolated using the ratio of isoflavone concentration at the 32nd hour to *k*_*e*_ (*C*_32_/*k*_*e*_). Total *AUC*_0–*∞*_ was the sum of *AUC*_0–32_ and *AUC*_32–*∞*_. The analysis for pharmacokinetic parameters was performed using the TOPFIT version 2.0 pharmacokinetic data analysis program for Windows.

#### 2.6.2. Statistical Analysis

The pharmacokinetic parameters were expressed as mean ± standard deviation (SD). The nonparametric Friedman's test was used to analyze the statistical differences among the mean values of pharmacokinetic parameters obtained following each of the three study phases. Differences were considered to be statistically significant if *P* value <0.05.

## 3. Results

Eleven healthy Thai postmenopausal women completed the study protocol. The average age of the participants was 58.09 ± 5.13 years. Their mean weight, height, and body mass index were 56.97 ± 3.95 kg, 1.55 ± 0.04 m, and 23.67 ± 0.81 kg/m^2^, respectively.

**Figure 2:**
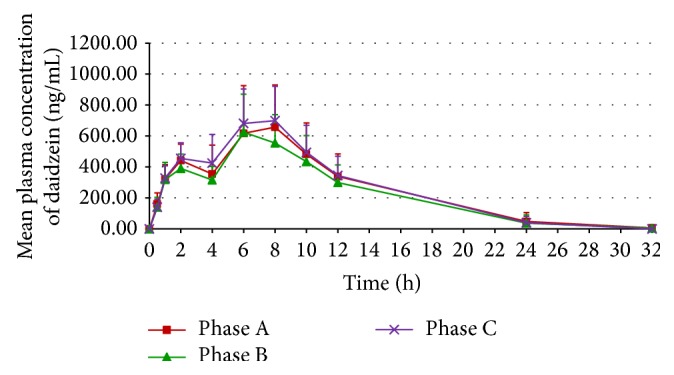
Mean plasma daidzein concentration-time curves from the 11 participants receiving a single oral dose of soy milk (Phase A), a single oral dose of soy milk coadministered with LWDHW (Phase B), and multiple oral doses of LWDHW for 14 days followed by a single oral dose of soy milk (Phase C). Error bars represent standard deviation (SD).

**Figure 3:**
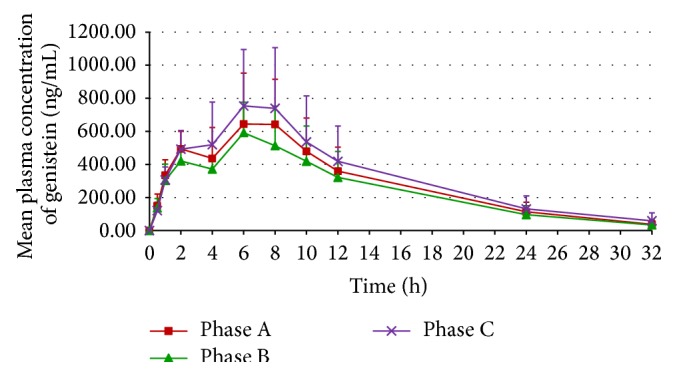
Mean plasma genistein concentration-time curves from the 11 participants receiving a single oral dose of soy milk (Phase A), a single oral dose of soy milk coadministered with LWDHW (Phase B), and multiple oral doses of LWDHW for 14 days followed by a single oral dose of soy milk (Phase C). Error bars represent standard deviation (SD).

The mean plasma concentration-time curves of daidzein and genistein from the 11 participants receiving the three regimens during the respective study phases are shown in Figures 2 and 3, respectively. The mean plasma concentration-time profiles of both daidzein and genistein obtained following each phase showed a biphasic pattern. Notably, the earlier peaks of both isoflavones occurred at approximately 2 hours, whereas the later peaks occurred at approximately 6–8 hours.

The pharmacokinetic parameters of daidzein and genistein in all participants obtained following each of the three study phases are demonstrated in Tables 1 and 2, respectively. The mean values of the pharmacokinetic parameters (*C*_max_, *T*_max_, *AUC*_0–32_, *AUC*_0–*∞*_, and *t*_1/2_) of daidzein and genistein did not statistically differ among the three study phases.

It is worth noting, however, that the mean values of *AUC*_0–32_ and *AUC*_0–*∞*_ of both daidzein and genistein obtained from Phase B were somewhat lower (approximately 10–15%) than those from Phase A, although the differences were not statistically significant. The mean values of* AUCs* obtained from Phase C, on the other hand, were somewhat greater than those of Phase A, although, again, not statistically significant. It is noteworthy, however, that this greater Phase C enhancement of* AUCs* than in Phase A was rather more pronounced for genistein than daidzein (approximately 15% versus 3%).

After the 14-day administration of LWDHW, the mean values of parameters used to assess safety (i.e., systolic and diastolic blood pressure, FSH level, complete blood count, and kidney and liver function) did not differ statistically or clinically from the baseline values (data not shown).

## 4. Discussion

The findings from this study revealed no statistically significant differences in the pharmacokinetic parameters of daidzein and genistein among the three regimens. However, the bioavailability of both isoflavone aglycones following coadministration of soy milk and LWDHW (Phase B) was somewhat lower than that of soy milk alone (Phase A), while the bioavailability of these aglycones after multiple-dose administration of LWDHW for 14 days (Phase C) tended to be greater than that of Phase A.

Soy milk, rather than other soy products such as soy extract capsules or other solid soy foods, was selected for this study because the isoflavones in soy milk are completely dissolved and ready to be absorbed, avoiding the need for investigation of disintegration and dissolution rates, factors which can affect isoflavone absorption via the gastrointestinal tract. The isoflavone dose used in this study was selected according to two main reasons. First, the recommended dose of isoflavones for relieving vasomotor symptoms in postmenopausal women is demonstrated to be approximately 50 mg/day or higher [6, 7, 21–23]. Second, it is generally accepted that doses of isoflavones ranging from 40 to 160 mg/day do not adversely affect endometrial thickness, vaginal cytology, or breast density [23–25]. Thus, the 90-mg total isoflavones dose used in the present study is well within the range of effective doses commonly used for relieving vasomotor symptoms in actual practice.

The generally recommended dose of LWDHW for relieving vasomotor symptoms (commonly equated to Yin Deficiency in Chinese medicine) ranges from 9 to 18 g/day [26] and can be consumed into two or three daily doses. Thus, the dose of 18 g/day used in this study is in accordance with the dose recommended in Chinese medical practice. Furthermore, the 14-day pretreatment with LWDHW in this study was appropriate for evaluation of its influence on isoflavone pharmacokinetics since previous studies have demonstrated that a 14-day pretreatment period is sufficient to affect the enzyme activities involved in Phase I (CYP1A2, CYP2A6, CYP3A4, CYP2C19, and CYP2D6) and Phase II (N-acetyltransferase2, NAT2) hepatic biotransformation. These studies have shown that LWDHW has no effect on CYP2C19, CYP2D6, and CYP3A4, whereas it can induce CYP1A2 but can suppress the activities of CYP2A6 and NAT2 [27, 28].

The mean plasma concentration-time curve profiles after a single oral dose of soy milk in this study exhibited a biphasic pattern, consistent with previously reported findings in Thai menopausal women [20, 29–31]. The appearance of the first peak, observed at approximately two hours after soy milk consumption, corresponds to the initial absorption of aglycones from the duodenum and proximal jejunum. During this step, lactase-phlorizin hydrolase and cytosolic *β*-glucosidase enzymes in the intestinal mucosa play an important role in the hydrolysis of isoflavone *β*-glycosides (i.e., daidzin and genistin, which are abundant isoflavone forms in soy milk) to their respective readily absorbable aglycones [32]. The second peak, occurring at approximately 6–8 hours, primarily reflects the capacity of gut microbiota to hydrolyze *β*-glycosides to their respective aglycones prior to absorption, which mainly occurs in the lower intestinal tract [33–35]. Additionally, the enterohepatic recirculation of isoflavones might also be partly involved in the occurrence of the second peak [34–36].

The findings from Phase B suggest that the bioavailability of both daidzein and genistein obtained following the coadministration of soy milk and LWDHW was approximately 10–15% lower than for soy milk alone (Phase A) despite the lack of statistical significance. The reduction in *C*_max_ of the first and second peaks of the plasma concentration-time curve (without any alternation in elimination *t*_1/2_) indicates that coadministration of LWDHW might negatively interfere somewhat with isoflavone absorption, either in the upper or lower gastrointestinal tract, or both. Since aglycones are readily absorbed by passive diffusion without any involvement with active or facilitated transport [37], LWDHW is unlikely to interfere with a carrier or the saturation process. A plausible explanation for this phenomenon is that some components in LWDHW might partially protect isoflavone *β*-glycosides from conversion to their respective readily absorbable aglycones. This possibility is in accordance with previous data showing that the composition of the food matrix (such as fiber and carbohydrates) can affect isoflavone bioavailability, especially in females [38]. Nonetheless, the impact of a single coadministered oral dose of LWDHW on isoflavone bioavailability seems negligible and should lack clinical significance.

The findings from Phase C, on the other hand, demonstrate a tendency for increased isoflavone bioavailability after pretreatment with multiple-dose LWDHW for 14 days compared to that with soy milk alone (Phase A). The increased isoflavone bioavailability in Phase C primarily correlated to an enhancement of *C*_max_ and* AUC* of the second peak, suggesting an impact of pretreatment with LWDHW on gut microbiota responsible for conversion of isoflavone *β*-glycosides to their respective absorbable aglycones as mentioned above. The underlying mechanism possibly involves the ability of pretreatment with LWDHW to stimulate the growth of *β*-glucosidase-producing microbiota (such as lactobacilli and bifidobacteria), especially in the lower gastrointestinal tract, yielding an enhanced hydrolysis of unabsorbable isoflavone *β*-glycosides to absorbable aglycone forms. This hypothesis is in accordance with previously reported data demonstrating that* Radix Rehmannia* (the main component of LWDHW) can exert a prebiotic effect via stimulation of* Lactobacilli* growth [39]. Nonetheless, the enhancement of* AUCs* caused by pretreatment with LWDHW in this study was rather more pronounced for genistein than daidzein, a finding which is in agreement with previous studies showing that prebiotic or probiotic supplementation enhances the absorption of genistein more than daidzein both in rats [40] and in human subjects [30, 41].

Taken together, the present study demonstrates that neither coadministration of single-dose LWDHW nor pretreatment with multiple-dose LWDHW significantly alters the pharmacokinetics and bioavailability of isoflavones. Therefore, concurrent use of LWDHW and isoflavones (from various soy products) is likely to be a simple and satisfactory method of enhancing drug compliance. Nonetheless, well-controlled clinical trials to investigate and verify the additive or synergistic efficacy of this combination should be conducted.

Some important limitations regarding the present study should be addressed. First, in order to fully avoid a carry-over effect of concurrent administration of LWDHW (conducted in Phases B and C) on isoflavone pharmacokinetics, this study was designed as a three-phase study with a fixed sequence separated by a washout period of at least one week rather than randomizing the sequences. Second, the sample size in this preliminary study was small. Further studies with larger samples to increase the statistical power to differentiate significant differences among study phases should be considered. Third, as this study did not include measurement of levels of isoflavones and their metabolites in urine and feces, the effects of LWDHW on isoflavone excretion was not investigated. Finally, this study focused on the influence of LWDHW on the pharmacokinetics of isoflavones, but it did not address the question of whether isoflavones affect the pharmacokinetics of LWDHW (or its active ingredients). Further investigation of those issues is warranted.

## 5. Conclusions

In healthy postmenopausal women, either coadministration of single-dose LWDHW or pretreatment with multiple-dose LWDHW for 14 days did not statistically alter the pharmacokinetic parameters of daidzein or genistein compared to those with the administration of a single oral dose of soy milk alone.

## Figures and Tables

**Table 1:** Pharmacokinetic parameters of daidzein obtained following each of the three study phases in the 11 participants enrolled in the study.

Pharmacokinetic parameters	Treatment phase			*P* value^*∗*^
	Phase A	Phase B	Phase C	
*C*_max⁡_ (ng/mL)	772.64 ± 247.30	715.45 ± 207.16	804.00 ± 173.03	0.06
*T*_max⁡_ (h)	6.55 ± 2.02	6.64 ± 2.29	6.55 ± 1.81	0.97
*AUC*_0–32_ (ng·h/mL)	7313.77 ± 3184.04	6234.05 ± 2536.89	7543.97 ± 2638.96	0.06
*AUC*_0–*∞*_ (ng·h/mL)	8169.06 ± 3056.75	7362.15 ± 2344.35	8416.20 ± 2522.09	0.18
*t*_1/2_ (h)	4.42 ± 0.88	4.58 ± 1.06	4.14 ± 0.80	0.81

^*∗*^Friedman's test.

Phase A, a single oral dose of soy milk; Phase B, a single oral dose of soy milk coadministered with LWDHW; Phase C, multiple oral doses of LWDHW for 14 days followed by a single oral dose of soy milk.

**Table 2:** Pharmacokinetic parameters of genistein obtained following each of the three study phases in the 11 participants enrolled in the study.

Pharmacokinetic parameters	Treatment phase			*P* value^*∗*^
	Phase A	Phase B	Phase C	
*C*_max⁡_ (ng/mL)	784.36 ± 371.67	670.36 ± 171.53	873.18 ± 336.34	0.09
*T*_max⁡_ (h)	5.36 ± 2.54	5.73 ± 2.28	6.00 ± 2.19	0.64
*AUC*_0–32_ (ng·h/mL)	9062.53 ± 5237.58	7871.89 ± 3054.41	10534.08 ± 4730.12	0.53
*AUC*_0–*∞*_ (ng·h/mL)	9956.56 ± 5441.06	8544.75 ± 3053.48	11291.46 ± 5030.50	0.53
*t*_1/2_ (h)	6.80 ± 0.62	6.69 ± 0.80	6.90 ± 0.89	0.91

^*∗*^Friedman's test.

Phase A, a single oral dose of soy milk; Phase B, a single oral dose of soy milk coadministered with LWDHW; Phase C, multiple oral doses of LWDHW for 14 days followed by a single oral dose of soy milk.
